# A promising method for the salvage of thrombosed native hemodialysis fistulas: percutaneous ultrasound-guided thrombolytic injection

**DOI:** 10.3906/sag-1902-206

**Published:** 2019-08-08

**Authors:** Hasanali DURMAZ, Erdem BİRGİ

**Affiliations:** 1 Department of Radiology, Dışkapı Yıldırım Beyazıt Training and Research Hospital, University of Health Sciences, Ankara Turkey

**Keywords:** Percutaneous, ultrasound, thrombolytic, native, hemodialysis fistulas

## Abstract

**Background/aim:**

It was aimed to describe the technical aspects and outcomes of percutaneous ultrasound-guided recanalization of thrombosed hemodialysis fistulas by thrombolytic injection.

**Materials and methods:**

A retrospective review was performed on patients with thrombosed native hemodialysis fistula who were treated using the percutaneous ultrasound-guided thrombolytic agent injection technique at the *interventional radiology* d*epartment*. A total of 17 patients [7 women (41.2%) and 10 men (58.8%)] were included in this study. All of the data, including demographic information and clinical findings, were obtained from the patients’ medical records and follow-up form of the procedure.

**Results:**

The mean fistula age was 5.6 years (range: 1–15 years). The mean diameter of the thrombosed segment was 5.53 cm (2–10 cm). Localization of the thrombi was in the aneurysmal segment at the level of needle insertion in 64.7% (n: 11) of patients, while it was on the venous side of the anastomosis in 35.3% (n: 6). The mean dose of tissue plasminogen activator (tPA) used in all of the sessions was 8.88 mg (5–17 mg). Overall technical success after all of the administrations was 100% and clinical success was 94.1%.

**Conclusion:**

Percutaneous ultrasound-guided thrombolytic injection in native hemodialysis fistulas is a rapid, practical, repeatable treatment method that is received on an outpatient basis with low risk of bleeding, and prevents unnecessary endovascular interventions or surgical operations.

## 1. Introduction

Chronic kidney disease (CKD) is an important health problem affecting people worldwide by virtue of high morbidity and mortality. Due to developing dialysis technology and therapies, the life-span and quality of life in patients undergoing hemodialysis with CKD is increased. Native arteriovenous fistula (AVF), synthetic loop grafts, and large-bore tunneled or temporary central venous catheters are the alternative hemodialysis methods for patients who cannot undergo renal transplant [1,2]. Among these options, native AVF is the first and most durable choice for vascular access because of the lower thrombotic and infectious complications [1,3]. 

Thrombosis is one of the most severe complications and is influential to morbidity and hospitalization in hemodialysis patients [4]. Diagnosis and endovascular treatment in the dysfunction of hemodialysis fistulae and grafts have been successfully performed by interventional radiologists over the last decades. Mechanical thrombectomy, pharmacomechanical thrombolysis, and pharmacologic thrombolysis via percutaneous infusion are currently being used for the management of hemodialysis vascular access [5].

The main objectives of endovascular/interventional procedures are to be minimally invasive, fast, and effective; thus, shortening the length of hospital stay and aiding in a quick return to daily life. Based on these objectives, a new technique for the treatment of thrombosed native hemodialysis fistulas was designed using an ultrasound, needle, and tissue plasminogen activator (tPA).

The purpose of this study was to evaluate the technical aspects and outcomes of percutaneous ultrasound-guided recanalization of thrombosed hemodialysis fistulas by thrombolytic agent injection.

## 2. Materials and methods

### 2.1. Patients

The medical records of patients who underwent percutaneous treatment for thrombosed hemodialysis fistulas were reviewed. A total of 17 patients [7 women (41.2%) and 10 men (58.8%)] were included in this study, from May 2012 to September 2017. Patients from hemodialysis centers and cardiovascular surgery with dysfunction of native hemodialysis fistulas were referred for the diagnosis and management of the recanalization. B-mode and Doppler ultrasonography (US) was used as the imaging method to detect the cause of dysfunction. None of the patients had a history of percutaneous transluminal angioplasty (PTA) or stent implantation due to stenosis or occlusion of a prior the hemodialysis fistula thrombosis. 

All of the data, including demographic information and clinical findings, were obtained from the patients’ medical records and follow-up form for the procedure (Table 1). The exclusion criteria for percutaneous treatment were an infected AVF, patients with hemostasis and bleeding disorders, the presence of chronic wall-adherent thrombi (>4 weeks old) and long segment thrombosis (>10 cm). Patients with nonthrombosed dysfunctional hemodialysis fistulas (low thrill) were also excluded from the study, as they were in need of endovascular fistulography.

**Table 1:** Patient demographic, AVF, and procedure-related data.

Patients
Female 7 41.2%
Male 10 58.8%
Mean age (range) 56.06 years (range: 32–77 years)
AVF types
End-to-side radiocephalic 12 70.6%
End-to-side brachiocephalic 3 17.6%
End-to-end radiocephalic 1 5.9%
End-to-side brachiobasilic 1 5.9%
Mean fistula age 5.6 years (range: 1–15 years)
Thrombus age
Acute (≤2 week old) 9 52.9%
Subacute (3–4 weeks old) 8 47.1%
Mean length of the thrombosed segment 5.53 cm (range: 2–10 cm)
The localization of thrombi
Aneurysmal segment 11 64.7%
Venous side of the anastomosis 6 35.3%
Mean dose of tPA (total of 20 sessions) 8.8 mg (range: 5–17 mg)
Mean dose of tPA (3 additional sessions) 5.6 mg (range: 5–7 mg)

Informed consent was obtained before initiating treatment and the ethics committee approved the study design.

### 2.2. Technique

In this study, the procedure was applied to outpatients without requiring hospitalization. Patients were primarily evaluated for the convenience of the procedure. For this purpose, patients referred from dialysis centers that were suspected of having thrombosis were evaluated with US.

**Figure 1:**
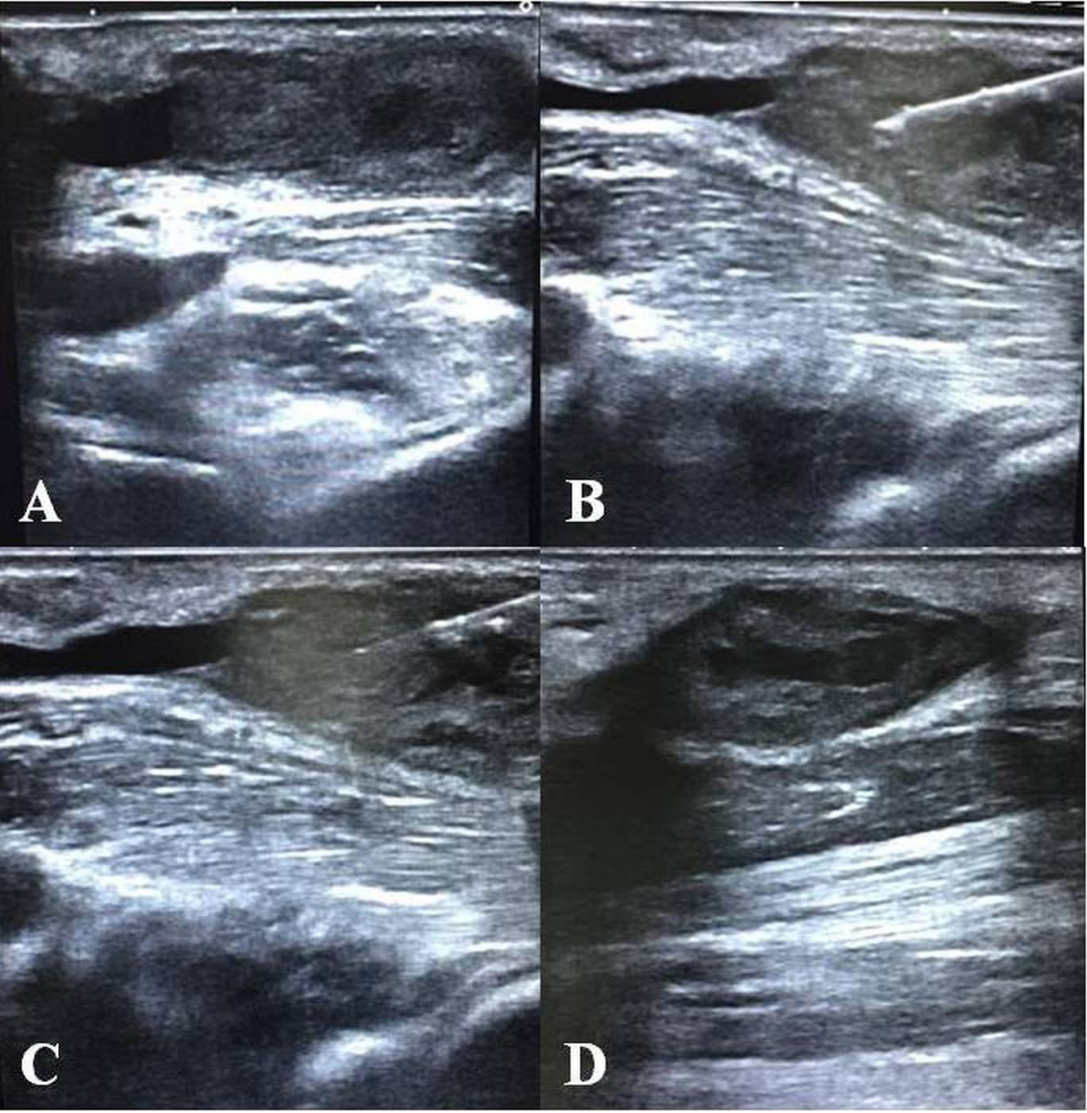
A 58-year-old male patient referred due to radiocephalic end-to-side native AV fistula dysfunction. The thrombus (A) detected at the level of the aneurysmal segment. A 27-G needle (B) was advanced percutaneously into the thrombus, and after tPA injection (C). Partial recanalization in the lumen was observed during the procedure (D).

**Figure 2:**
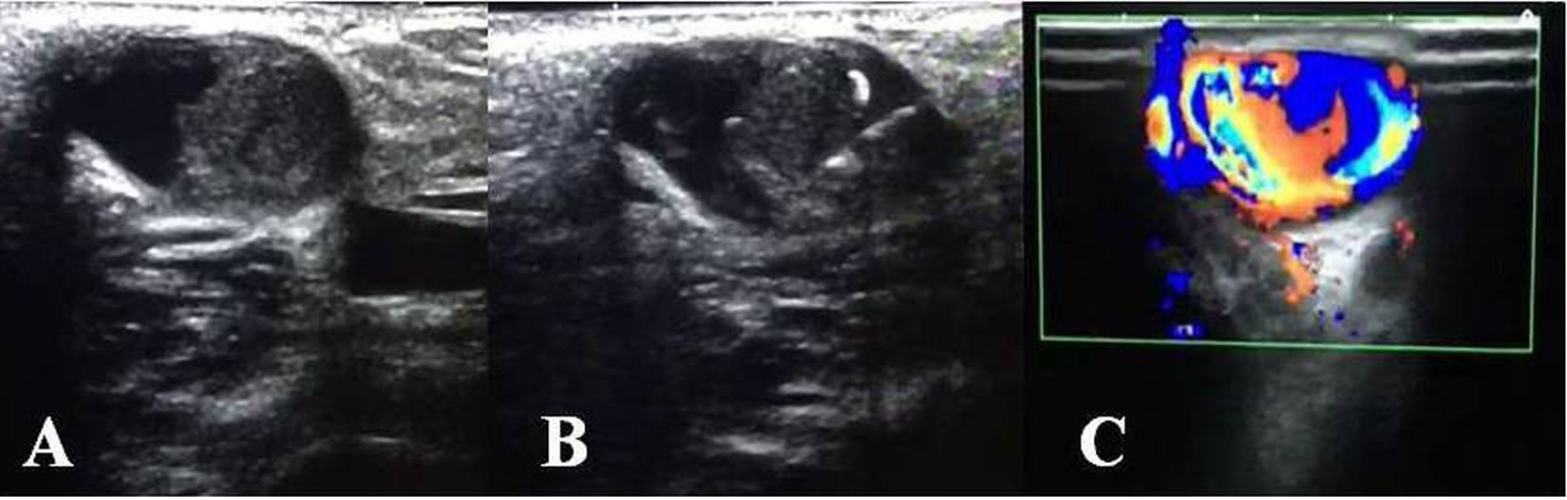
Thrombus at the site of an aneurysmal segment in a 72-year-old male patient with brachiocephalic end-to-side fistula is seen (A). Percutaneous ultrasound-guided tPA injection was performed (B). Doppler ultrasound revealed the complete patency in the lumen at the first day check-up (C).

Thrombus on the venous side of a hemodialysis fistula that restricted the flow or completely led to occlusion, with a sonographic view suggesting acute and subacute stage, in patients with a clinical history of less than 4 weeks and coagulation parameters within normal limits (INR of <1.5, PLT of >50,000/mm3) were accepted for treatment. The length, location, and nature of the thrombosis and fistula characteristics were recorded using Doppler ultrasound. After providing appropriate sterile conditions, using a 27-gauge (G) needle with a 5-cc conventional syringe, 5–10 mg of tPA was percutaneously injected into different localizations of the thrombus by entering different points of the skin. Particular attention was paid to the administration of the tPA into the thrombus, and the procedure was performed in a controlled manner under US guidance (Loqic S6, GE Healthcare, USA). Local anesthesia was provided at the puncture sites with 1% lidocaine. No prophylactic antibiotic was administered. In most patients, the thrombus started to dissolve during the injections (Figure 1). After each procedure, the arteries and veins associated with the fistula were evaluated by Doppler US. If the central veins could not be evaluated with Doppler US, fistulography was performed in order to see the underlying pathology. After tPA administration, the patients were advised to massage this area for approximately 10–15 min every hour during the day to facilitate homogenous spread of the tPA to the thrombosed segment. The patients were kept under observation for approximately 2 h for close follow-up of any complications after the procedure. The patients were sent home 2 h after that and called the next day to check their lumen patency (Figure 2). An additional dose of percutaneous tPA (mean: 5.6 mg, range: 5–7 mg) was administered to patients who did not have sufficient patency at their check-up. Patients who received an additional dose were then again called for a check-up after 1 day. In the US check-ups performed after the first or second session, postprocedure flow-volume rates (700–1300 mL/min) were measured by Doppler US****to evaluate the adequate patency for sufficient dialysis [6]. Otherwise, patients needed fistulography to determine additional pathologies. The use of an anticoagulant was recommended in those patients to reduce the risk of recurrent thrombosis. Patient follow-ups were made by contacting their dialysis centers and getting information about the status of their dialysis sessions after the procedure. In the case of the recurrence of missed dialysis sessions, the patients were redirected to us and checked by dialysis fistulography to identify additional pathologies that led to the thrombosis. Patients with stenosis or occlusions at the anastomosis, efferent vein, or central vein were treated via endovascular methods.

**Table 2:** Success and patency rates of the treatment.

Overall technical success	100%
Clinical success	94.1%
Primary patency rate	82.3%
Secondary patency rate	76.5%

### 2.3. Definitions

An aneurysmal segment was defined as dilation twice that of the normal draining vein or dilation that was >3 cm in diameter. Technical success was defined as <30% residual diameter in stenosis after treatment. Clinical success after the thrombolytic injection was defined as the resumption of normal dialysis for at least 2 sessions. Primary patency was defined as the interval following the thrombolytic injection until insufficient lumen patency. Secondary patency was defined as the endovascular treatment of the central vein or outflow draining vein by angioplasty, with or without stent implantation. Complications were categorized as major or minor. Major complications included those requiring therapy or hospitalization, causing permanent adverse sequelae, or death. Minor complications were those requiring no or nominal therapy [7].

### 2.4. Statistical analysis

Numeric data were presented as the average, standard deviation, median, maximum, and minimum, while categorical data were pre­sented as the number and percentage. Statistical data editing and analysis were per­formed using SPSS (IBM Corp., Armonk, NY, USA) v.20.0 software.

## 3. Results

A total of 17 patients with thrombosed native hemodialysis fistulas (7 women and 10 men; mean age: 56.06 years, range: 32–77 years, SD 12.96 years) were included in the study. The patients were mainly referred from dialysis centers due to failure of a dialysis session. The types of AVFs were end-to-side radiocephalic (n: 12, rate: 70.6%), end-to-side brachiocephalic (n: 3, rate: 17.6%), end-to-end radiocephalic (n: 1, rate: 5.9%), and end-to-side brachiobasilic (n: 1, rate: 5.9%). AVFs were located on the right upper limb in 7 patients and on the left in 10 patients. The mean fistula age was 5.6 years (range: 1–15 years).

Intravenous clots treated by percutaneous ultrasound-guided thrombolytic injection (≤4 weeks old) were divided into 2 subgroups, as acute (≤2 weeks old) and subacute (3–4 weeks old), 52.9% (n: 9) and 47.1% (n: 8), respectively. The mean length of the thrombosed segment was 5.53 cm (range: 2–10 cm, SD: 2.26 cm). Localization of thrombi was in the aneurysmal segment at the level of needle insertion in 64.7% (n: 11) of patients, while it was on the venous side of the anastomosis in 35.3% (n: 6). The degree of thrombosis was total in 9 patients (52.9%) and partial in 8 patients (47.1%). Considering of all of the patients, 20 sessions of percutaneous ultrasound-guided thrombolytic injection procedure were performed and a total of 151 mg of tPA was used. The mean dose of tPA used in all of the sessions was 8.88 mg (range: 5–17 mg, SD: 3.95 mg) (Table 1).

Central venous catheterization is known to be a risk factor for central vein stenosis or occlusion. In 3 patients (17.7%), there was no medical history of catheter insertion. In 14 patients (82.3%), there was a history of permanent or temporary dialysis catheter insertion in the side of fistula or counter. 

Overall, the technical success after all of the administrations was 100% and clinical success was 94.1% (16 of 17 patients). In 3 patients (17.7%), insufficient lumen patency was detected, thus a second session of thrombolytic injection was needed on the first day check-up. In 3 patients, after 2 sessions of thrombolytic injection, and in 1 patient, after the diagnosis of underlying stenosis in the outflow draining vein, endovascular treatment was needed, after a mean number of 3.7 dialysis sessions. As the endovascular treatment, PTA for the efferent vein stenosis in 2 patients, PTA for the central vein stenosis in 1 patient, and PTA with stenting for central vein stenosis in 1 patient was performed due to repetition of the AVF dysfunction. The primary patency rate was 82.3% and secondary patency rate was 76.5% (Table 2).

Minor hemorrhage was resolved in 1 patient by manual compression. During and after the procedures, no major complications were encountered.

## 4. Discussion

In hemodialysis patients, early recognition and treatment of complications like thrombosis is important for long-term and effective use of the fistula. Thrombosis is a frequent complication in native hemodialysis fistulas and the underlying pathology is mostly an aneurysmatic segment causing flow turbulences and stenosis in the outflow or central vein [8]. In addition to outflow pathologies, hypotension, low cardiac output, reduction in the arterial flow due to stenosis, and clotting problems can lead to thrombosis [9]. Native AVF thromboses are often recognized by insufficient flow during routine dialysis sessions, as in the current study. Dialysis staff may refer a patient with complaints of cannulation difficulty or aspirating clot. This patient needs immediate hemodialysis, and therefore must undergo the placement of a temporary dialysis catheter until the declotting procedure. Conventional treatments for AV fistula deficiencies include surgical thrombectomy, new shunting in the proximal side of the old AVF, patch plasty or bridge graft procedures with autogenous or synthetic grafts, and arterial or venous bypass procedures. Although the long-term results of these procedures are acceptable, they are not preferred for reasons such as being more invasive than endovascular treatments and not allowing early cannulation; thus, temporary dialysis catheterization is needed [10].

Endovascular procedures in interventional radiology have increased in number thanks to the rapid development of equipment and expanding field of imaging. The main techniques used for the declotting of AVFs in interventional radiology are catheter-directed thrombolysis, catheter-directed mechanical thrombectomy. and angioplasty, with or without stenting [11]. 

Considering the workload in interventional radiology departments and the necessity of continuity of dialysis sessions for their hemodialysis patients, the quest for effective, practical, and rapid treatment methods, for both the practitioner and patient, gave rise to the idea of percutaneous ultrasound-guided thrombolytic injection for recanalization of thrombosed native hemodialysis fistulas. Using this method,****thromboses that led to missed dialysis sessions were treated using only an ultrasound, needle, and tPA. This technique was chosen specifically for thromboses not longer than 10 cm, as it was believed that a thrombolytic infusion catheter would be more effective in long segments of thrombosis.

Aside from the difficulty of placing the thrombolytic infusion catheter in the thrombus and fixing the catheter to this point in short segment fistula thrombi, due to the use of a long infusion catheter, the risk of hemorrhagic complications may be increased because some of the given tPA will pass into the systemic circulation outside of the thrombosed segment. Herein, the tPA dose injected percutaneously was 5–10 mg mean per session and it was administered only locally into the thrombus; thus, the risk of bleeding complications was significantly lower due to the lack of transition to the systemic circulation and was thought to have completed its half-life by acting within the thrombus. 

Thanks to this technique, the thrombus at the site of the dialysis cannulation was treated successfully in 76.5% (n: 13/17) of the patients, thus prevented from unnecessary endovascular intervention as well as surgery. Moreover, an average number of 3.7 dialysis session continuity was maintained in 4 patients that subsequently needed endovascular intervention due to outflow vein stenosis. The advantage of percutaneous treatment in those 4 patients was to reduce the burden of thrombus and allow us to visualize the underlying stenosis by fistulography. Moreover, the use of fluoroscopy, owing to the ultrasound-guided technique, in proper cases was reduced. Flow-volume rates of the AVFs were measured using Doppler US after the procedure to show the adequate patency for dialysis. In the event of low flow-volume rates despite the lysis of the thrombus, fistulography was needed to show the outflow pathologies.

The retrospective design of the study was the main limitation, in addition to the study group being limited in number.

As a conclusion, percutaneous ultrasound-guided thrombolytic injection in native hemodialysis fistulas is a rapid (approximately 15–30 min), practical, repeatable treatment method, received on an outpatient basis with low risk of bleeding that prevents unnecessary endovascular interventions or surgical operations. Since this technique has not been described previously in the literature, we believe that a comparison of endovascular methods and the percutaneous technique described herein should be performed with larger patient groups.
